# Breastfeeding indicators trends in Brazil for three decades

**DOI:** 10.11606/S1518-8787.2017051000029

**Published:** 2017-11-13

**Authors:** Cristiano Siqueira Boccolini, Patricia de Moraes Mello Boccolini, Fernanda Ramos Monteiro, Sonia Isoyama Venâncio, Elsa Regina Justo Giugliani

**Affiliations:** I Laboratório de Informações em Saúde. Instituto de Comunicação e Informação Científica e Tecnologia em Saúde. Fundação Oswaldo Cruz. Rio de Janeiro, RJ, Brasil; II Faculdade de Medicina de Petrópolis. Faculdade Arthur Sá Earp Neto. Petropolis, RJ, Brasil; III Coordenadora Nacional das Políticas de Aleitamento Materno. Departamento de Ações Programáticas Estratégicas. Ministério da Saúde. Brasília, DF, Brasil; IV Instituto de Saúde. Secretaria de Estado da Saúde de São Paulo. São Paulo, SP, Brasil; V Departamento de Pediatria. Faculdade de Medicina. Universidade Federal do Rio Grande do Sul. Porto Alegre, RS, Brasil

**Keywords:** Breast Feeding, trends, Time Series Studies, Health Surveys, Aleitamento Materno, tendências, Estudos de Séries Temporais, Inquéritos Epidemiológicos

## Abstract

**OBJECTIVE:**

Update breastfeeding indicators trend in Brazil for the last three decades, incorporating more up-to-date information from the National Health Survey.

**METHODS:**

We used secondary data from national surveys with information on breastfeeding (1986, 1996, 2006, and 2013) to construct the time series of prevalence for the following indicators: exclusive breastfeeding in children under six months of age (EBF6m), breastfeeding in toddlers under 2 years of age (BF), continued breastfeeding at one year of age (BF1year), and continued breastfeeding at two years of age (BF2years).

**RESULTS:**

The prevalence of EBF6m, BF, and BF1year increased until 2006 (rising from 4.7%, 37.4%, and 25.5% in 1986 to 37.1%, 56.3%, and 47.2% in 2006, respectively). For these three indicators, there was relative stabilization between 2006 and 2013 (36.6%, 52.1%, and 45.4%, respectively). The BF2years indicator had a distinct behavior – relatively stable prevalence, around 25% between 1986 and 2006, and a subsequent increase, reaching 31.8% in 2013.

**CONCLUSIONS:**

The time series of breastfeeding indicators in Brazil shows an upward trend until 2006, stabilizing from that date onwards on three of the four indicators evaluated. This result, which can be considered as a warning sign, requires evaluation and revision of policies and programs to promote, protect and support breastfeeding, strengthening existing ones and proposing new strategies so that the prevalence of breastfeeding indicators returns to an upwards trend.

## RESUMO

**OBJETIVO:**

Atualizar a tendência dos indicadores de aleitamento materno no Brasil nas últimas três décadas, incorporando informações mais recentes provenientes da Pesquisa Nacional de Saúde.

**MÉTODOS:**

Utilizamos dados secundários dos inquéritos nacionais com informações sobre aleitamento materno (1986, 1996, 2006 e 2013) para a construção da série histórica das prevalências dos seguintes indicadores: aleitamento materno exclusivo em menores de seis meses de vida (AME6m), aleitamento materno em menores de dois anos (AM), aleitamento materno continuado com um ano de vida (AM1ano) e aleitamento materno continuado aos dois anos (AM2anos).

**RESULTADOS:**

As prevalências de AME6m, AM e AM1ano tiveram tendência ascendente até 2006 (aumentando de 4,7%, 37,4% e 25,5% em 1986 para 37,1%, 56,3% e 47,2% em 2006, respectivamente). Para esses três indicadores, houve relativa estabilização entre 2006 e 2013 (36,6%, 52,1% e 45,4%, respectivamente). O indicador AM2anos teve comportamento distinto – prevalência relativamente estável, em torno de 25% entre 1986 e 2006, e aumento subsequente, chegando a 31,8% em 2013.

**CONCLUSÕES:**

A série histórica dos indicadores de aleitamento materno no Brasil mostra tendência ascendente até 2006, com estabilização a partir dessa data em três dos quatro indicadores avaliados. Esse resultado, que pode ser considerado um sinal de alerta, impõe avaliação e revisão das políticas e programas de promoção, proteção e apoio ao aleitamento materno, fortalecendo as existentes e propondo novas estratégias para que as prevalências dos indicadores de aleitamento materno retomem a tendência ascendente.

## INTRODUCTION

Breastfeeding offers countless benefits for children and women and is the intervention with the greatest potential to reduce infant mortality^5^. Optimal levels of breastfeeding could prevent more than 820,000 deaths of children under five years old per year worldwide, as well as prevent 20,000 deaths of women by breast cancer^17^. A recent systematic review conducted by Victora et al.^17^ reaffirms the protection that breastfeeding offers against infectious diseases and a lower risk of dental malocclusion and chronic diseases (such as diabetes and overweight in breastfed children), as well as its impact on a better performance in intelligence tests^17^. It is believed that the increase in the prevalence and duration of breastfeeding observed since the 1970s^15^ contributed significantly to the improvement in child health indicators in Brazil^17^, reducing, for example, hospitalizations due to diarrhea and respiratory infections in children under one year of age in the country^1^ ^,^ ^2^.

In the 1970s, there was a “weaning epidemic”, due to the intense urbanization process, the entry of women into the labor market, and the unregulated advertising and marketing of industrialized milk throughout the world^15^. As a reaction to this fact, Brazil created the National Breastfeeding Program (PNBF) in 1981, distinguished by its diversity of actions, among which: regulation of the commercialization of infant food, implementation of the Hospital Amigo Criança^10^, the creation of the Brazilian Network of Human Milk Banks^3^, the adoption of the Kangaroo Method^4^ as public policy, the implementation of the *Amamenta e Alimenta Brasil* Strategy^8^, and, more recently, the inclusion of actions aimed at working women who breastfeed. Because of this policy, national surveys conducted since 1975 have shown an expansion of the practice of exclusive breastfeeding in children between zero and six months of age and increased median duration of breastfeeding, in line with WHO recommendations^15^.

The National Health Survey (PNS), conducted in 2013, is the latest population-based health survey conducted in Brazil^12^, with information on breastfeeding practice, which allows for a renewed analysis of the breastfeeding trend in the country. Thus, the objective of this study was to update the breastfeeding indicators trend in Brazil for the last three decades, incorporating more up-to-date information from the PNS.

## METHODS

### Study Design and Data Sources

A time trend study with secondary data from national population-based surveys conducted in 1986^a^, 1996^b^, 2006,^c^ and 2013^12^, to chart the breastfeeding practice trend in Brazil.

The National Survey on Maternal and Child Health and Family Planning (PNSMIPF-1986) resulted from a subsample of the National Household Sample Survey (1984), with a probabilistic sample in two stages and representative of the six geographic regions of the study period (Rio de Janeiro, São Paulo, South, East Central, and Federal District, Northeast, and North and Midwest (the latter two aggregated), and excluded from the sample the rural areas of the North and Midwest regions, the population of the state of Acre and the territories of Rondônia, Roraima, and Amapá). In that research, 8,519 households were selected, with 6,733 women aged 15–44 years old were interviewed^a^.

The National Survey on Demography and Health of 1996 (PNDS-1986) comprised a sub-sample of the National Household Sample Survey (PNAD-1995), with a probabilistic sample in two stages, representative of the seven PNAD regions of the study period – Rio de Janeiro, São Paulo, South, East Central, Northeast, North (urban areas), and Midwest. Data from 13,283 households and 12,612 women aged 15–49 were obtained and these women provided information about their children who were under five years of age (n = 4,782 children)^b^.

The National Survey of Demography and Health of Children and Women (PNDS-2006) was carried out by two-stage probabilistic sampling, based on the Demographic Census of 2000, stratified by the five major regions (North, Northeast, Midwest, South, and Southeast) and by the situation of the household (urban and rural), totaling 10 strata. A total of 14,617 households were visited, and information on 15,575 women aged 15–49 years old and their respective children under five years of age (n = 5,461) was obtained^d^.

Finally, the National Health Survey of 2013 (PNS-2013) used a sample plan by clusters in three stages of selection, with a resident aged 18 years or older being interviewed in each household (with equiprobability among all adult residents of the household), based on the master sample of the Brazilian Institute of Geography and Statistics (IBGE). There were 64,348 home interviews and 60,202 individual interviews with the resident selected at home. Of these individuals, 31,845 were women. Of these women, 4,215 had children under two years of age, who were considered for the present study^15^.

The PNSMIPF and PNDS-1986 were part of the Demographic and Health Surveys (DHS) Program, with public data obtained with prior authorization from DHS, through the website www.measuredhs.com (Accessed on March 20, 2016). The PNDS-2006, although using the DHS criteria and questionnaires, was not part of the DHS research group of this period, and its micro data were obtained from the website www.bvsms.saude.gov.br/bvs/pnds/banco_dados.php (Accessed on Dec 20, 2015). It is worth mentioning that, while PNSMIPF and PNDS were designed to evaluate aspects related to maternal and child health, the focus of the PNS-2013 was the health situation of the adult population in general. The data of the PNS-2013 were obtained through the IBGE website, http://www.ibge.gov.br/home/estatistica/populacao/pns/2013/default_microdados.shtm (Accessed on Dec 21, 2015).

### Inclusion and Exclusion Criteria

For PNSMIPF-1986 and PNDS 1996 and 2006, children under two years of age were selected at the time of the interview and non-living children were excluded at the time of the interview; among twins, only the first-born child was selected for the study, and if there was more than one child in that age group in the same household, only the youngest child was considered for the analysis.

For PNS-2013, we included children under two years of age residing in the selected households at the time of the interview, whose information was obtained from the mothers or guardians of the children; if there was more than one child in this age group at home or twins, only the youngest participated in the study.

### Questionnaires

In order to obtain information on breastfeeding, mothers/guardians were questioned about the availability of breast milk for their children within 24 hours prior to the interview in all surveys. Information was also obtained about other foods consumed in the last 24 hours: in the 1986 survey, they were questioned about the consumption of water, juice, powdered milk, cow’s milk, other liquids and solid or soft food; in the 1996 survey, information was obtained on water consumption (pure or with sugar), fruit juice, tea or herbs, milk powder, infant formula, fresh milk (pure or with water), other liquids, yogurt, industrialized soft food, soft food with flour, soft food with rice, soft food with vegetables, eggs, fish, chicken or beef; in 2006, they were questioned about the consumption of water (pure or with sugar), fruit juice, tea, milk powder, fresh milk (pure or with water), other liquids, soft foods, multi-mixture, dairy foods or family food; and in the PNS-2013, data was obtained on the consumption of other milks (and derivatives), water, tea, porridge, fruit or natural fruit juice, artificial juices, vegetables and legumes, beans (different types of beans); meats or eggs, potatoes and other tubers and roots, biscuits, crackers and cakes, sweets and candies, soft drinks and other foods.

### Indicators

The definition of exclusive breastfeeding (EBF) adopted in this study follows the 2007 recommendations by WHO^e^, i.e. the child should receive “only breast milk (directly from the breast or pumped) and no other liquid or solid (except for medicines, mineral supplements or vitamins)”. For the construction of the “EBF” variable, the woman should have offered breast milk in the last 24 hours and offered no other food or liquid listed in each survey.

Indicators related to the prevalence of breastfeeding were calculated based on the WHO recommendation (WHO, 2008). The prevalence of EBF in children less than six months of age (EBF<6m) was obtained according to the proportion of children between zero and five full months of life who were exclusively breastfed.

Other indicators used were the prevalence of BF (BF – proportion of children born in the last two years who were breastfed at any time in the last 24 hours); prevalence of continued BF in the first year of life (BF1year – proportion of children between 12 and 15 months of age breastfed at the time of the interview) and prevalence of continued BF at 2 years of age (BF2years – proportion of children between 20 and 23 months who were being breastfed at the time of the interview). As the available PNS-2013 tabulation did not allow direct comparability with the indicators proposed by the WHO, it was necessary to modify the BF1year indicator for 12 to 14 months of age, and the BF2years indicator for 21 to 23 months of age. It was not possible to compare the prevalence of breastfed children and breastfeeding in the first hour of life since this information was not included in the PNS-2013.

### Statistical Analyses

The point prevalence and 95% confidence intervals of the EBF<4m, EBF<6m, BF, BF1year, and BF2years indicators were estimated, considering the complex sample design of each survey^13^ and the stratification by age group of the child at the moment of the interview.

### Ethical Considerations

Because this information is publicly accessible, without access restrictions for researchers and citizens in general, and not subject to limitations related to privacy, security or access control, this study did not require prior approval by the Ethics Committee, in accordance with Brazilian Ethics Resolution 466/12^f^.

## RESULTS

Regarding the indicators used, the prevalence of EBF among children under six months increased by 34.2 percentage points between 1986 and 2006, going from 2.9% to 37.1%, with statistically significant gains in each decade until 2006 and stabilization in 2013. A similar pattern was observed with the prevalence of BF, which increased statistically by 18.9 percentage points between 1986 and 2006, reaching a prevalence of 56.3% in 2006. However, in 2013 there was a slight decrease in BF (52.1%). The prevalence of BF in the first year of life increased from 22.7% in 1986 to 45.4% in 2013, equivalent to a total increase of 22.7 percentage points in the period, stabilizing between 2006 and 2013. The evolution of the prevalence of BF at two years of age differed from other indicators, with a relatively stable prevalence of around 25% between 1986 and 2006, and a subsequent statistically significant increase of 8.5 percentage points, reaching 31.8% in 2013 (Table 1).

**Table 1:** Prevalence of breastfeeding in Brazil, by the national survey, between 1986 and 2013.

Year	1986^a^	1996^b^	2006^c^	2013^d^
				
Characteristic	Prevalence (95%CI)^h^	Prevalence (95%CI)^h^	Prevalence (95%CI)^e^	Prevalence (95%CI)^e^
EBF<4m^f^	4.7 (1.7–12.0)	29.2 (24.0–35.0)	45.0 (35.7–54.6)	-
EBF<6m^g^	2.9 (1.1–7.4)	23.9 (19.8–28.5)	37.1 (29.7–45.2)	36.6 (30.4–42.9)
BF^h^	37.4 (31.5–43.6)	44.8 (42.2–47.4)	56.3 (52.4–60.1)	52.1 (50.0–54.2)
BF1year^i^	22.7 (12.9–36.8)	37.5 (31.1–44.2)	47.2 (36.5–58.2)	45.4 (39.4–51.3)
BF2years^j^	24.5 (11.7–44.4)	24.7 (20.0–30.2)	23.3 (15.2–33.9)	31.8 (25.4–38.1)

^a^ National Survey on Maternal and Child Health and Family Planning of 1986.

^b^ National Demography and Health Survey of 1996.

^c^ National Survey on Demography and Children and Women’s Health of 2006.

^d^ National Health Survey of 2013.

^e^ Prevalence (95%CI): Point prevalence and 95% confidence interval estimated considering the complex design of the sample.

^f^ EBF<4m: prevalence of exclusive breastfeeding among children younger than 4 months of age.

^g^ EBF<6m: prevalence of exclusive breastfeeding among children younger than 6 months of age.

^h^ BF: prevalence of breastfeeding among children younger than 24 months of age.

^i^ BF1year: prevalence of breastfeeding among children 12 to 14 months of age.

^j^ BF2years: prevalence of breastfeeding among children 21 to 23 months of age.

The prevalence of EBF decreased with increasing age. Between 1986 and 2006, there was a 44% increase in the prevalence of EBF among infants from zero to two months of age and of 28.1% among infants of three to five months. In 2013, there was a 0.3% reduction in the prevalence of EBF of among infants from zero to two months of age and of 15.1 percentage points among babies three to five months of age in relation to the 2006 survey (Table 2).

**Table 2:** Prevalence of exclusive breastfeeding in children under 24 months of age in Brazil, by age and population survey, from 1986 to 2013.

Age (months)	Year				Period			
	
	1986^a^	1996^b^	2006^c^	2013^d^	1986/1996 (10 years)^f^	1996/2006 (10 years)^f^	2006/2013 (7 years)^f^	1986/2013 (27 years)^f^
	Prevalence (%)^e^				Difference (%)^f^			
0–2	6.0	42.4	50.0	49.7	36.4	7.6	-0.3	43.7
3–5	1.6	12.6	29.7	14.6	11.0	17.1	-15.1	13.0

^a^ National Survey on Maternal and Child Health and Family Planning of 1986.

^b^ National Demography and Health Survey of 1996.

^c^ National Survey on Demography and Children and Women’s Health of 2006.

^d^ National Health Survey of 2013.

^e^ Prevalence of exclusive breastfeeding calculated by the current status method, with smoothed estimates by moving averages, according to the method proposed by Segall-Corrêa et al.c (2009).

^f^ Absolute difference calculated by the difference between the prevalence of exclusive breastfeeding.

The Figure shows the continuous increase in the prevalence of BF up to 18 months from 1986 to 2006, with the highest gain in the age groups between two and 19 months. The curve for the year 2013, when compared to 2006, showed a decline in the prevalence of breastfeeding in the first 12 months of age. The prevalence of breastfeeding from the age of 21–23 months, which had been stagnant since the first survey, increased in the 2013 survey.

**Figure:**
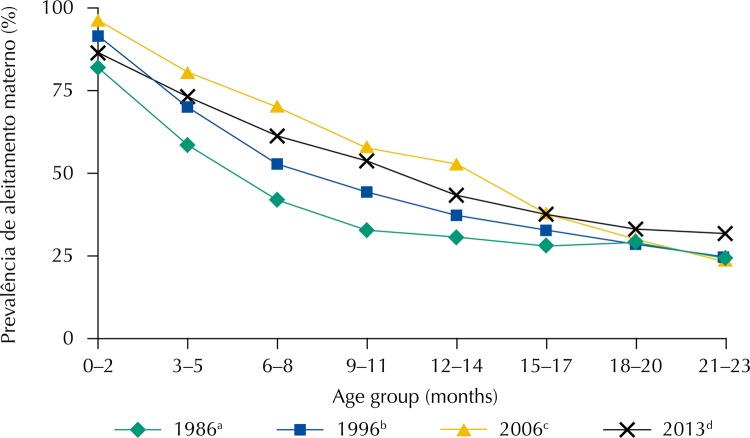
Prevalence of breastfeeding, by age group and the national survey. Brazil, 1986^a^, 1996^b^, 2006^c^ and 2013^d^.

^a^ Series 1 – National Survey on Maternal and Child Health and Family Planning of 1986.

^b^ Series 2 – National Demography and Health Survey of 1996.

^c^ Series 3 – National Survey on Demography and Children and Women’s Health of 2006.

^d^ Series 4 – National Health Survey of 2013.

## DISCUSSION

In the last three decades, the prevalence of breastfeeding and exclusive breastfeeding indicators in Brazil presented an upward trend, with the main gains observed between 1986 and 2006, followed by relative stabilization in 2013. On the other hand, continued breastfeeding until the second year remained stable between 1986 and 2006, being the only indicator with an increase in prevalence between 2006 and 2013.

Previous studies have found an increase in the median duration of breastfeeding in national surveys, ranging from 2.5 months in 1975^15^ to 14 months in 2006^16^, and in surveys involving only Brazilian capitals and the Federal District (from 9.9 months in 1999 to 11.9 months in 2008)^14^. These data led Brazil to be considered a successful country in the implementation of breastfeeding promotion policies and programs, which reached the three distinct levels of intervention proposed by Rollins et al.^11^: individual levels (characteristics and relationships between mothers and babies), local (health services, family and community, and place of work and employment), and structural (sociocultural and marketing characteristics). This success has been largely attributed to the regulation and monitoring of the commercialization of infant food, the adoption of the Baby Friendly Hospital Initiative and the Mother-Kangaroo Strategy^4^, to the creation and expansion of the coverage of the Human Milk Banks^3^ and the implementation of the *Amamenta e Alimenta Brasil* Strategy (related to primary care level)^8^, as well as the adoption of labor laws that extended paid maternity leave from four to six months, optionally and subsidized by the government.

Pérez-Escamilla et al.^9^ conducted a systematic review that proposed the evaluation of BF policies using the “gears” model, in order to facilitate the adoption of strategies at the national level and to ensure the sustainability of breastfeeding programs and policies. The authors identified that Brazil has all the necessary gears for the advancement of breastfeeding indicators: political will, legislation and policies, financial resources, training and teaching in policies and programs, breastfeeding promotion, research and evaluation, advocacy, and a central coordination with objectives and monitoring^9^, indicating that the success of breastfeeding is not the sole responsibility of women but is shared by all society^11^.

The results of PNS-2013 do not take away Brazil’s merit for having been recognized internationally as a successful country in promoting, protecting, and supporting breastfeeding in the two publications cited^9^ ^,^ ^11^. However, it’s necessary to reflect on these results. The deceleration in 2013 of the gains that had been observed between 1986 and 2006 is worrying; for the first time in the time series no real gains were observed in the prevalence of breastfeeding, the most troubling decrease being EBF among children three to five months of age.

It is natural that the greater the gains in the prevalence of breastfeeding, the greater the difficulties to continue to increase them^14^. However, the levels of breastfeeding indicators, despite the significant increase over time, still far short of the recommendations on the duration of breastfeeding and EBF. According to the WHO criteria, Brazil is only in a reasonable position regarding the prevalence of exclusive breastfeeding in children under six months and in a poor position in terms of duration of breastfeeding^14^.

Considering the national scenario of breastfeeding indicators deceleration, a critical evaluation of all the actions currently under way in Brazil is necessary, as follows: agreement on a public policy to encourage breastfeeding by the States and Cities; expansion of the number of hospitals certified in the Baby Friendly Hospital Initiative (including the accreditation of private hospitals); and expansion of the *Amamenta e Alimenta Brasil* Strategy and other actions aimed at primary health care (such as breastfeeding support and human milk collection rooms). In addition, it is necessary to extend maternity leave to six months for all workers; broad monitoring of Law 11.265/06, which regulates the marketing of foods and human milk substitutes for infants; and extension of the activities of the Human Milk Banks towards the primary health care network. It is also important to consider other aspects, such as the valorization of human resources for the implementation and monitoring of actions to promote, protect, and support breastfeeding, strengthen spaces and political actors supporting breastfeeding, regional cultures, different labor occupations, different lifestyles, vulnerable and minority populations (such as the indigenous population and prison populations), and increased participation of the father, when present, and of the family in the breastfeeding process. The decrease in the prevalence of exclusive breastfeeding between three and five months of age between 2006 and 2013 confirms the need to reinforce strategic actions to support working women during the lactation period.

On the other hand, it is important to highlight the increase, for the first time in the time series, of the prevalence of breastfeeding at the child’s 18 months of age forward. This may seem contradictory; however, it has been shown that the determinants of continued breastfeeding in the first year of life may be different from those for maintaining breastfeeding as recommended by the WHO, i.e. for two years or more. For example, the mother cohabiting with a partner protects breastfeeding in the first year^7^ but is an inhibitor for the maintenance of breastfeeding after two years of age or more^6^.

The main limitations of the study are due to methodological differences between surveys. The standardized surveys by Demographic and Health Surveys in 1986, 1996, and 2006 allow comparison among themselves when interviewing only women of childbearing age (15–49 years). PNS-2013, although also a national survey, followed a different methodology when interviewing adults 18 years of age or older, and responses about breastfeeding may be obtained from persons other than the mother of the child. To reduce this bias and allow comparability, we only selected children whose mothers or guardians were interviewed.

It is important to note that the indicator that suffered the most modifications during the surveys was exclusive breastfeeding. The following may have contributed to this: the difference in the food list included in the questionnaires of the different surveys, the list becoming increasingly complex and reaching a higher number of questions in the 2013 survey; the increase in the EBF recommended period (from four to six months up to 2001^18^ to six months thereafter (WHO, 2007)^g^; the difference in the formulation of the question that defined the duration of the EBF – in the 1996 survey, when the mother said she fed the child only breast milk, questions about water and tea consumption in the last 24 hours were not applied, which precludes a reliable analysis of exclusive breastfeeding practice in this survey.

Another aspect worth mentioning is the lack of disaggregated data in the PNS-2013, making it difficult to compare the prevalence of EBF among children less than four months of age. In addition, the age ranges available for breastfeeding infants under one year and two years of age were different for the surveys from 1986 to 2006 and the PNS-2013 (12 to 15 months *versus* 12 to 14 months, and 20 to 23 months *versus* 21 to 23 months, respectively), and it is necessary to adopt age groups slightly different from those proposed by the WHO. These differences tend to overestimate the prevalence of BF1year and to underestimate the prevalence of BF2years in the PNS-2013, since in the former case, the group consisted of younger children and, in the second, older children.

We believe that the PNS-2013 data can be used for comparison purposes with surveys from 1986 to 2006, considering the above limitations. A new edition of the DHS national survey, originally scheduled for 2016, would be instrumental in confirming the trends presented here. In addition, it is necessary to conduct future studies that consider the distribution of the individual characteristics of mothers and babies over the decades, since these characteristics can influence the rates of breastfeeding and EBF.

In conclusion, we believe that the participation of the government, civil society and class entities in the mobilization of society and government were important for the advancement of breastfeeding rates observed between 1986 and 2006. However, there is a warning sign in the lack of progress, for the first time in the time series of national surveys, of the prevalence of breastfeeding and EBF observed in the PNS-2013, compared to PNDS-2006, since even with methodological differences, it was expected that this prevalence should increase. These results reinforce the need to intensify the actions already implemented and develop new actions to promote, protect and support breastfeeding, involving the various sectors of society, in order to resume the growth of the prevalence and duration of breastfeeding. On the other hand, the increase in the prevalence of breastfeeding from the age of 18 months, which had been stagnant since the beginning of the time series, should be commemorated. An in-depth analysis of this phenomenon may aid in the return of the increase of all breastfeeding indicators.

## Footnotes

a Arruda JM, Rutenberg N, Morris L, Ferraz EA. Pesquisa Nacional sobre Saúde Naterno-Infantil e Planejamento Familiar-PNSMIPF-Brasil, 1986. Rio de Janeiro: BEMFAM; 1987 [cited 2017 Apr 27]. Available from: https://dhsprogram.com/pubs/pdf/FR4/FR4.pdf

b Sociedade Civil Bem Estar Familiar no Brasil; Macro International; Institute for Resource Development. Demographic, and Health Surveys. Brasil: Pesquisa Nacional sobre Demografia e Saúde - 1996. Rio de Janeiro; 1997 [cited 2017 Apr 27]. Available from: https://dhsprogram.com/pubs/pdf/FR77/FR77.pdf

c Ministério da Saúde (BR), Centro Brasileiro de Análise e Planejamento. Pesquisa Nacional de Demografia e Saúde da Criança e da Mulher PNDS 2006. Brasília (DF); 2009 [cited 2017 Apr 27]. p.196-209. (Série G. Estatística e Informação em Saúde). Available from: http://bvsms.saude.gov.br/bvs/publicacoes/pnds_crianca_mulher.pdf

d Cavenaghi S. Aspectos metodológicos e comparabilidade com pesquisas anteriores. In: Ministério da Saúde, Centro Brasileiro de Análise e Planejamento. Pesquisa Nacional de Demografia e Saúde da Criança e da Mulher- PNDS 2006: dimensões do processo reprodutivo e da saúde da criança. Brasília (DF); 2009 [cited 2017 Apr 27]. p.14-32. (Série G. Estatística e Informação em Saúde). Available from: http://bvsms.saude.gov.br/bvs/publicacoes/pnds_crianca_mulher.pdf

e World Health Organization. Indicators for assessing infant and young child feeding practices: part 1: definitions: conclusions of a consensus meeting held 6-8 November 2007 in Washington DC, USA. Geneva; 2008 [cited 2017 Apr 27]. Available from: http://apps.who.int/iris/bitstream/10665/438 95/1/9789241596664_eng.pdf

f Ministério da Saúde (BR), Conselho Nacional de Saúde. Resolução Nº 466, de 12 de dezembro de 2012. Aprova diretrizes e normas regulamentadoras de pesquisas envolvendo seres humanos. Brasília (DF); 2012 [cited 2017 Apr 27]. Available from: http://bvsms.saude.gov.br/bvs/saudelegis/cns/2013/res0466_12_12_2012.html

g World Health Organization. Indicators for assessing infant and young child feeding practices. Part 1: definitions: conclusions of a consensus meeting held 6-8 November 2007 in Washington, DC, USA. Geneva: WHO; 2007 [cited 2017 Apr 27]. Available from: http://apps.who.int/iris/bitstream/10665/43895/1/9789 241596664_eng.pdf
